# Insulin Activation Mediated by Uptake Mechanisms: A Comparison of the Behavior between Polymer Nanoparticles and Extracellular Vesicles in 3D Liver Tissues

**DOI:** 10.1021/acs.biomac.3c00102

**Published:** 2023-04-06

**Authors:** Angela Costagliola di Polidoro, Zahra Baghbantarghdari, Vincenza De Gregorio, Simona Silvestri, Paolo Antonio Netti, Enza Torino

**Affiliations:** † Interdisciplinary Research Centre on Biomaterials (CRIB), University of Naples Federico II, P.le Tecchio 80, Naples 80125, Italy; ‡ Department of Chemical, Materials and Production Engineering (DICMaPI), University of Naples Federico II, P.le Tecchio 80, Naples 80125, Italy; § Fondazione Istituto Italiano di Tecnologia, IIT, Largo Barsanti e Matteucci 53, Naples 80125, Italy; ∥ Department of Biology, University of Naples ″Federico II″, Complesso Universitario di Monte S Angelo, Naples 80125, Italy

## Abstract

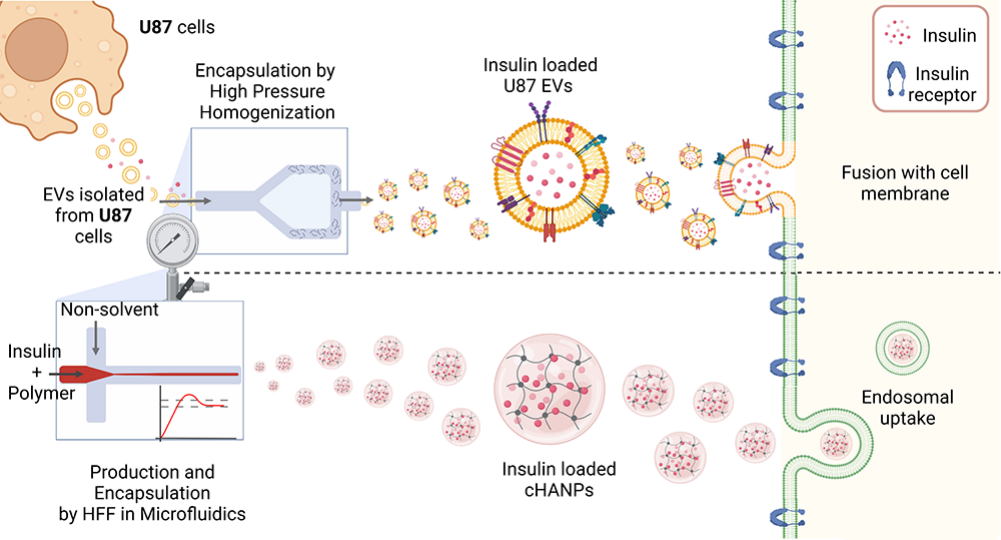

In this work, we compare the role of two different uptake mechanisms in the effectiveness of a nanoformulated drug, specifically insulin. Insulin is activated by interacting with insulin receptors exposed on the liver cell membrane that triggers the uptake and storage of glucose. To prove that the uptake mechanism of a delivery system can interfere directly with the effectiveness of the delivered drug, two extremely different delivery systems are tested. In detail, hydrogel-based NPs (cHANPs) and natural lipid vesicles (EVs) encapsulating insulin are used to trigger the activation of this hormone in 3D liver microtissues (μTs) based on their different uptake mechanisms. Results demonstrated that the fusion mechanism of Ins-EVs mediates faster and more pronounced insulin activation with respect to the endocytic mechanism of Ins-cHANPs. Indeed, the fusion causes an increased reduction in glucose concentration in the culture medium EV-treated l-μTs with respect to free insulin-treated tissues. The same effect is not observed for Ins-cHANPs that, taken up by endocytosis, can only equal the reduction in glucose concentration produced by free insulin in 48 h. Overall, these results demonstrate that the effectiveness of nanoformulated drugs depends on the identity they acquire in the biological context (biological identity). Indeed, the nanoparticle (NP) biological identity, such as the uptake mechanism, triggers a unique set of nano-bio-interactions that is ultimately responsible for their fate both in the extracellular and intracellular compartments.

## 1 Introduction

When nanoparticles (NPs) come in contact with the biological environment, every biological identity gives rise to a specific set of nanotechnology to biology (*i.e.*, nano-bio) interactions that determine a unique structure–activity relation specific to the formulation.^1^ When not adequately understood, characterized, and controlled, this relation can severely limit the effectiveness of a nanoformulation regardless of its diagnostic and therapeutic potential.^2^

Although some achievements have been accomplished in the design of nanosized architectures,^3−8^ the evaluation of nano-bio-interactions to control their effectiveness in a relevant biological environment is still a fundamental challenge of nanomedicine.^1^ This is particularly true if considering, for example, the impact that the uptake mechanism triggered by a specific nanoparticle design can have in the delivery and release of drugs and thus directly on their activation.

In this regard, Wilhelm et al.^9^ collected and compared more than 200 studies on preclinical testing of NPs, revealing the impact that each single NP characteristic (composition, size, shape, surface charge, and surface functionalization) has on the delivery efficiency of the analyzed formulations, defined as the percentage of the dose that accumulates in the target site with respect to the administered one. Interestingly, results showed that each NP characteristic influences the delivery efficiency of a formulation to a different extent depending on the formulation itself. Thus, the knowledge developed around a specific nanoparticle-based system cannot be generalized to other nanosized vectors.

Our work investigates the role of optimal design of nanocarriers, comparing polymer-based nanoparticles and lipid-based vesicles to accomplish a specific biological task, the delivery of insulin in 2D and 3D *in* vitro models. The observation in the same specific biological context of these differently designed nanovectors aims to widen the comprehension of the connection between the synthetic and biological identities potentially linked to the prediction of their pharmacokinetic profile *in vivo*, NP stability, diffusion, and cargo release to tissue and target cell uptake.

In detail, we explore the role of cross-linked hyaluronic acid nanoparticles (cHANPs), a full polymer, hydrogel-based nanoparticle, and extracellular vesicles (EVs) derived from U87 cells both encapsulating insulin as a model drug. They are independently analyzed in mediating the activation of this hormone as a consequence of their uptake in vitro on 2D adherent cell and tissue diffusion and on 3D tissue models of Hep-G2 liver microtissues (l-μTs). In this sense, literature has widely reviewed uptake mechanisms revealing that generally, hydrogel nanoparticles, based on the particle’s composition, size, charge, and surface functionalization follow vesicle-based endocytosis (e.g., clathrin and caveolin-mediated uptake, receptor-mediated endocytosis, micropinocytosis) in many different cell lines.^7,10−13^ On the contrary, there is evidence that the uptake of EVs occurs by fusion with the recipient cell membrane.^14−16^ In fusion, direct contact between the two lipid bilayers in proximity primarily causes a fusion stalk and then its expansion into a diaphragm bilayer, allowing the formation of a pore where the two hydrophobic cores are mixed.^17^

Therefore, selecting the hormone insulin as a model drug allows identifying the difference in the regulation of glucose concentration in the l-μT culture medium induced by the two different uptake mechanisms. Indeed, the regulation of hepatic glucose production and its therapeutic effect depends on a signaling cascade that originates from the interaction of insulin with its receptors exposed on the cell membrane of hepatocytes.^18^

## 2 Experimental Section

Hyaluronic acid HA (Mw = 50,000 Da) is purchased from Creative PEGWorks. Divinyl sulfone (DVS) also known as vinyl sulfone (contains <650 ppm hydroquinone as the inhibitor; purity 97%; density 1117 g/mL at 25 °C (lit.); Mw = 118.15 g/mol), gelatin powder (type B, Mw 176,654 Da), acetone (CHROMASOLV, for HPLC, ≥99,8%; molecular formula CH_3_COCH_3_; Mw = 58.08), ethanol (ACS reagent, ≥99.5% (200 proof), absolute; molecular formula CH_3_CH_2_OH; Mw = 46.07), sodium hydroxide (NaOH) (ACS reagent, ≥97.0%, Mw = 40.00), Tween 85, Span 85, and ATTO 633 (MW 652 g/mol–Ex/Em 633/655) are purc hased from Merck KGaA (Germany). A PKH67 green fluorescent cell linker kit for general cell membrane labeling Em/Ex 490/502, MW = 652 g/mol, and insulin (solution 10 mM), Dulbecco’s modified Eagle medium, high glucose (DMEM), fetal bovine serum (FBS), phosphate buffer saline (PBS), trypsin, penicillin/streptomycin, and l-glutamine, for cell culture and in vitro study are purchased by Sigma-Aldrich. Nonessential amino acids (NEAA) and sodium pyruvate are purchased from GIBCO. Minimum Essential Medium (MEM) Earle’s salt is purchased from Microtech. A sieve shaker (SieveShaker IG/3-EXP) is purchased from Giuliani Tecnologie srl. U87 MG and Hep-G2 cell lines are purchased from ATCC (ATCC, Milan, Italy). The water used for synthesis and characterization is purified by distillation, deionization, reverse osmosis (Milli-Q Plus), and filtered with a 0.22 μm cutoff.

### 2.1 Cross-Linked Hyaluronic Acid Nanoparticle (cHANP) Production and Insulin Encapsulation

cHANP production is a consolidated process.^7,8,19^ Briefly, nanoparticles are produced in a microfluidic X-junction chip where the hydrodynamic flow focusing regime is used to implement a nanoprecipitation process. To produce cHANPs loaded with insulin, the solvent phase is made of HA (0.5% w/V) and insulin (0.1 mg/mL), and it is fed into the middle channel at 27 μL/min. A pH equal to 12.3 is obtained by adding NaOH to promote the cross-linking reaction between −OH groups of HA and divinyl sulfone (DVS). The aqueous phase is made of acetone and DVS (4% V/V) and fed into the side channels at 110 μL/min. The sample is collected in 20 mL of acetone and wheel-stirred overnight to promote the completion of the cross-linking reaction by DVS diffusion. Non-encapsulated insulin is purified by a two-step solvent gradient dialysis, dropping the suspension in a Spectra Por Cellulose Membrane 6 (MWCO 50 kDa). Increasing gradients of acetone–ethanol (70/30% V/V, 50/50% V/V, and 30/70% V/V) in the first phase and ethanol–water gradients in the same ratio for the second phase are used. Each phase has a duration of 1 h. Continuous magnetic stirring at 230 rpm and room temperature is used to promote diffusion.

### 2.2 Extracellular Vesicle (EV) Isolation and Insulin Encapsulation

EVs (EVs) are isolated from U87 cells, as previously reported.^20^ Briefly, cells are seeded in a T150 flask at a density of 4 × 10^6^ and allowed to grow until 70–80% confluency. Then, the culture medium is replaced with a fetal bovine serum-free culture medium (Thermo Fisher Scientific, Waltham, MA), and cells are left to grow for 48 h. Thus, for isolation, 50 mL of EV-rich culture medium is collected and transferred to centrifuge polypropylene tubes. Consequent centrifugation steps are used to isolate EVs, all performed at 4 °C. In the first step, the collected EV-rich culture medium is centrifuged for 5 min at 500*g* (F15-6x 100y ROTOR), then at 2000*g* for 10 min using an SL 16R Centrifuge, and finally 30 min at 10,000*g* to remove dead cells and cell debris. As a final step, EVs are ultracentrifuged and separated from the supernatant by differential ultracentrifugation (dUC) in 8 mL of polycarbonate tubes (Beckman Coulter, Brea, CA) at 110,000*g* (70,000 RPM–MLA-80 ROTOR) for 70 min using an Optima MAX-XP ultracentrifuge (Beckman Coulter, Brea, CA). The supernatant is discarded, and the pellet is resuspended in 200 μL of PBS.

Insulin encapsulation in EVs is performed through high-pressure homogenization (HPH), as previously reported.^20^ Briefly, 17.5 mL of PBS containing 0.6 × 10^11^ particles and insulin (100 μM) are processed in a Y-type fixed-geometry interaction chamber (diamond F20 Y-75 μm chamber) of a high-pressure homogenizer bench-top microfluidizer (model M-110P Microfluidizer Materials Processor, Microfluidics, Westwood, MA) at 500 bar for five cycles, assuring the preservation of the insulin structure and EV integrity. The stability of encapsulated insulin is assessed by circular dichroism (J-815 spectropolarimeter, Jasco, Tokyo, Japan). A circular dichroism spectrophotometer is used for secondary structure studies (180–250 nm) and measures are performed on 200 μL of the sample setting CD to 20 mdeg/0.05 dOD, FL scale to 20 mdeg/0.05 dOD, D.I.T at 0.5 s, bandwidth 1nm, and data pitch at 0.5 nm. During the process, a thermocouple is used to monitor the temperature in the reservoir close to the discharge port to avoid fluctuations. An ice bath to decrease the temperature in the external cooling coil is used to maintain the temperature in a range from 4 to 10 °C. EV integrity after encapsulation is assessed indirectly, based on the amount of proteins in the volume through a BCA assay (QuantiPro BCA Assay Kit, Sigma-Aldrich), performed according to the vendor’s instructions. Excess insulin is removed by centrifugation inside a 100 kDa spin Corning (Spin-X-μF concentrators, Corning) at 1000 rpm, 10 °C for 25 min.

### 2.3 Chemical–Physical Characterization of EVs and cHANPs

NP size, polydispersity, and superficial charge are measured by dynamic light scattering (DLS, Zetasizer Nano ZS, Malvern U.K.). Typically, 1 mL of the diluted sample is put in 12 mm square glass cuvettes for 90° sizing (Optical Cuvette, Sarstedt) and measured at least in triplicate. ζ potential measurements are performed at room temperature on a Zetasizer Nano ZS (Malvern, U.K.), fitted with a high-concentration ζ potential cell. Nanoparticle morphology is assessed by scanning and transmission electron microscopy, SEM and TEM, respectively. For SEM observations (Carl Zeiss Ultraplus Field Emission), 100 μL of purified samples are dropped on a polycarbonate isopore membrane filter (cutoff 0.05 μm) under vacuum ultrafiltration and let dry overnight. Before the observation, 5–7 nm of Au is deposited on the sample. For cryo-TEM observation of EVs (Cryo-TEM TECNAI by FEI), 5 μL of the purified sample is dropped on a Formvar/Carbon 200 mesh grid (Agar scientific) and observed at an 80 kV accelerating voltage. The concentration of cHANPs and EVs is quantified by nanoparticle tracking analysis NTA (Nanosight NS300, Malvern Instruments Ltd., U.K.). Both samples are observed at a dilution of 1:100, at 25 °C for 300 s with a manual shutter and gain adjustment. Results are analyzed with NP Tracking Analysis software.

### 2.4 Determination of NP Encapsulation Efficiency (EE%)

To quantify the insulin content successfully encapsulated in cHANPs, purified NPs are disrupted, and the liberated drug is quantified by BCA assay. NPs are swollen by the addition of NaOH to the particle suspension until reaching a pH of 13 and let react overnight. Disrupted cHANPs are then filtered with a 0.22 μm filter and the filtered sample is analyzed with a spectrophotometer.

Insulin encapsulation efficiency (EE%) is calculated as
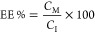
where *C*_M_ is the concentration measured in the lysed sample, and *C*_I_ is its initial concentration.

Differently, for EVs, insulin is quantified indirectly by quantification of the purified insulin from the encapsulated sample through the BCA assay. Indirect encapsulation efficiency (EE%) is calculated as
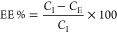
where *C*_E_ is the excess of insulin purified from the encapsulated sample, and *C*_I_ is its initial concentration. We used an indirect measurement for the evaluation of the insulin to preserve its structure, very unstable if exposed to Triton X 100 needed to perform the direct measurement by digestion of the vesicles.

### 2.5 Uptake of NPs by Adherent Hep-G2 Cells

Uptake of NPs by hepatocellular carcinoma (Hep-G2) adherent cells is studied to investigate the characteristic time of NP/cell interaction for both tested systems. For this purpose, 50000 cells/well are plated in a 48-well plate (Falcon) and filled with 0.5 mL of culture medium (Minimum Essential Medium Earle’s Salt (MEM, Microtech), containing 10% FBS, 100 μg mL^–1^l-glutamine, 100U mL^–1^ penicillin/streptomycin, 0.1 mM nonessential amino acid, and 0.1 mM sodium pyruvate). Cells are left to adhere for 24 h and, then, exposed to fluorescently labeled EVs (2.15 × 10^10^ particles/mL) or cHANPs (0.6 × 10^10^ particles/mL), both dispersed in FBS-free culture medium to avoid interferences with EV internalization. Specifically, EVs are stained with the green dye PKH67, diluting one EV sample in PBS and, according to the protocol recommended by the producer, mixed with the diluted dye. The suspension is rotated on the wheel for 5 min to ensure homogeneity. The sample is then ultracentrifuged in 1.5 mL polyallomer tubes (Beckman Coulter, Brea, CA) at 100,000*g*, 4 °C (MLA-150 ROTOR) for 70 min to remove the excess dye. The supernatant is discarded, and the pellet is resuspended in PBS. Differently, a NIR standard dye, ATTO 633, is encapsulated in cHANPs through microfluidics, as previously reported.^7^

After 6, 8, 12, and 24 h contact with NPs, cells are washed three times with PBS to remove noninternalized particles, trypsinized, and resuspended in 250 μL of fresh culture medium. NP uptake is quantified by single-cell-fluorescence detection by flow cytometry measurements (BD FACS Celesta, Becton, Dickinson and Company, New Jersey). Additionally, side scattering and forward scattering measurements are recorded as an indication of cell granularity and viability.^21−23^

### 2.6 Three-Dimensional Liver Microtissue (3D Liver-μT) Production and Histological Assessment

Gelatin-porous microbeads (GPMs) are prepared according to a slightly adapted double emulsion (O/W/O) protocol.^24^ Briefly, 10 mL of water containing TWEEN 85 (6% w/v) is used to dissolve the gelatin powder (type B, Mw 176,654 Da, Sigma-Aldrich) at 60 °C. Then, to produce oil-in-water (O/W) emulsion, a solution of toluene and SPAN 85 (3% w/v) is added to the aqueous gelatin solution (8% w/v) (Sigma-Aldrich). To obtain GPMs, 30 mL of additional toluene is added (O/W/O) and cooled to 5 °C. Lastly, to achieve toluene extraction and gelatin microbead stabilization, ethanol (20 mL) is poured. The GPMs are, then, left to dry under a chemical hood overnight and sieved (sieve shaker IG/3-EXP, Giuliani Tecnologie srl) to obtain microscaffolds of 75–150 μm in diameter. Afterward, the GPMs are stabilized with glyceraldehyde at 4%, as previously described.^25^ In order to produce 3D liver-μTs, Hep-G2 are cultured in Minimum Essential Medium Earle’s Salt (MEM, Microtech), containing 10% fetal bovine serum, 100 μg mL^–1^l-glutamine, 100U mL^–1^ penicillin/streptomycin, 0.1 mM nonessential amino acid, and 0.1 mM sodium pyruvate. Cells are, then, incubated at 37 °C in a humidified atmosphere (5% CO_2_). Dried GPMs are sterilized with ethanol and washed with PBS, and 35 mg of GPMs are loaded with 5.25 × 10^6^ cells (30 cell/GPM ratio) in a bioreactor spinner flask in dynamic conditions (CELLSPIN, 250ml, Integra Biosciences). In particular, to promote the epithelial cell seeding on GPMs, an intermittent stirring regime (30 min at 0 rpm, 5 min at 30 rpm) is used for 24 h. Afterward, the stirring speed is kept at a continuous 20 rpm and maintained at 37 °C in a humidified 5% CO_2_ incubator for up to 5–7 days. The culture medium is changed three times per week. For histological analysis, 1 mL aliquots of 3D liver-μTs, collected at day 7, were fixed in a 10% neutral buffered formalin solution for 1 h, rinsed in PBS, dehydrated in an incremental series of alcohol (75, 85, 95, and 100% twice, each step 30 min at RT), then treated with xylene (30 min twice), and embedded in paraffin. 5 μm thick tissue sections were stained with hematoxylin and eosin according to the manufacturer’s protocol, mounted on coverslips with Histomount mounting solution (Bioptica), and observed with a light microscope.

### 2.7 Study of Nanoparticle Penetration in 3D Liver-μTs

Three-dimensional diffusion of NPs in 3D liver-μTs is observed to highlight different NP interactions and tissue penetration. 3D liver-μTs are withdrawn from a spinner flask, filtered with a homemade polycarbonate sieve of 350 micron meshes to select microtissues, and loaded into 96-well plates (4–5 liver-μTs/well). All samples are assayed in triplicate. The samples are transferred onto a μ-Slide 8 Well glass bottom (IBIDI) and analyzed under a confocal microscope to monitor the uptake and penetration of the particles in real time. cHANPs (1.2 × 10^10^ particles/mL) and EVs (6 × 10^10^ particles/mL) are added to the suspension medium without FBS and left to interact for 24 or 48 h. Untreated 3D liver-μTs and cultured in serum-free-medium are used as a negative control.

Confocal imaging is performed with an HCX PL APO CS 63 × 1.40 oil objective. An argon laser at 488 nm and a HeNe 633 nm laser are used. Z-stacks up to 45 μm depth into the tissues to understand NP tissue penetration are acquired. Quantification of NP penetration is performed through total image fluorescence quantification by ImageJ. Briefly, total fluorescence is acquired for each z-step, which has different thicknesses depending on the local thickness of the observed tissues. This value is corrected for autofluorescence by subtracting the fluorescence of the untreated tissues with the same laser settings. These normalized values are then plotted against tissue thickness and the fluorescence profile is analyzed.

### 2.8 Insulin Therapeutic Efficacy on 3D Liver-μTs

The role that each tested drug delivery system and its synthetic identity have in controlling the interaction with 3D liver-μTs and thus on the therapeutic effect of insulin is investigated by measuring the concentration of glucose in the culture medium of μTs exposed to the same concentration of insulin (35 μM) in different formulations (free insulin, insulin encapsulated in cHANPs, insulin encapsulated in EVs). Glucose consumption by differently treated μTs at different time points (24 and 48 h) is quantified through a glucose assay kit by Thermo Fisher (EIAGLUC, glucose test) according to the vendor’s instructions. A glucose standard at different concentrations (0.5–16 mg/dL) is used to build a standard curve. Samples are measured at different dilutions to fit the standard curve concentrations at least in triplicate.

### 2.9 Statistical Analysis

Data are reported as the mean ± standard error of the mean (SEM). Statistical analyses are performed with one-way ANOVA followed by Tukey’s test for multiple comparisons of differently treated groups (control, free Ins, Ins-cHANPs, Ins-EVs). *P* values <0.5 denote statistically significant differences. In detail (*) denotes *P* values <0.5, (**) *P* values <0.1, and (***) *P* values <0.05, NS indicates no significant differences.

## 3 Results and Discussion

### 3.1 cHANPs and EVs: The Synthetic Identity of Nanoparticles

Composition, size, surface charge of NPs, and loaded active agents are some attributes contributing to defining the synthetic identity of a nanoparticle-based drug delivery system.^26^

In this work, two highly different systems, characterized by different synthetic identities, are developed and let to perform the same biological task, the delivery of insulin to liver cells. The rationale of the study is to investigate the set of complex cell-nanoparticle nano-bio-interactions raised by these different systems in the biological environment and their impact on insulin activity.

First, a full polymer, hydrogel-based nanocarrier and second, a lipid natural vesicle are loaded separately with insulin, a model of a membrane-interacting drug that needs to be available to its receptors to trigger the signaling cascade responsible for its therapeutic effect.^27^

For both systems, encapsulation occurs by the exploitation of highly controlled microfluidic processes, a low-pressure process for polymer nanoparticles, and a high-pressure process for natural vesicles.

This section presents the physical–chemical characterization and insulin encapsulation efficiency (EE%) of the two drug delivery systems.

**Figure 1:**
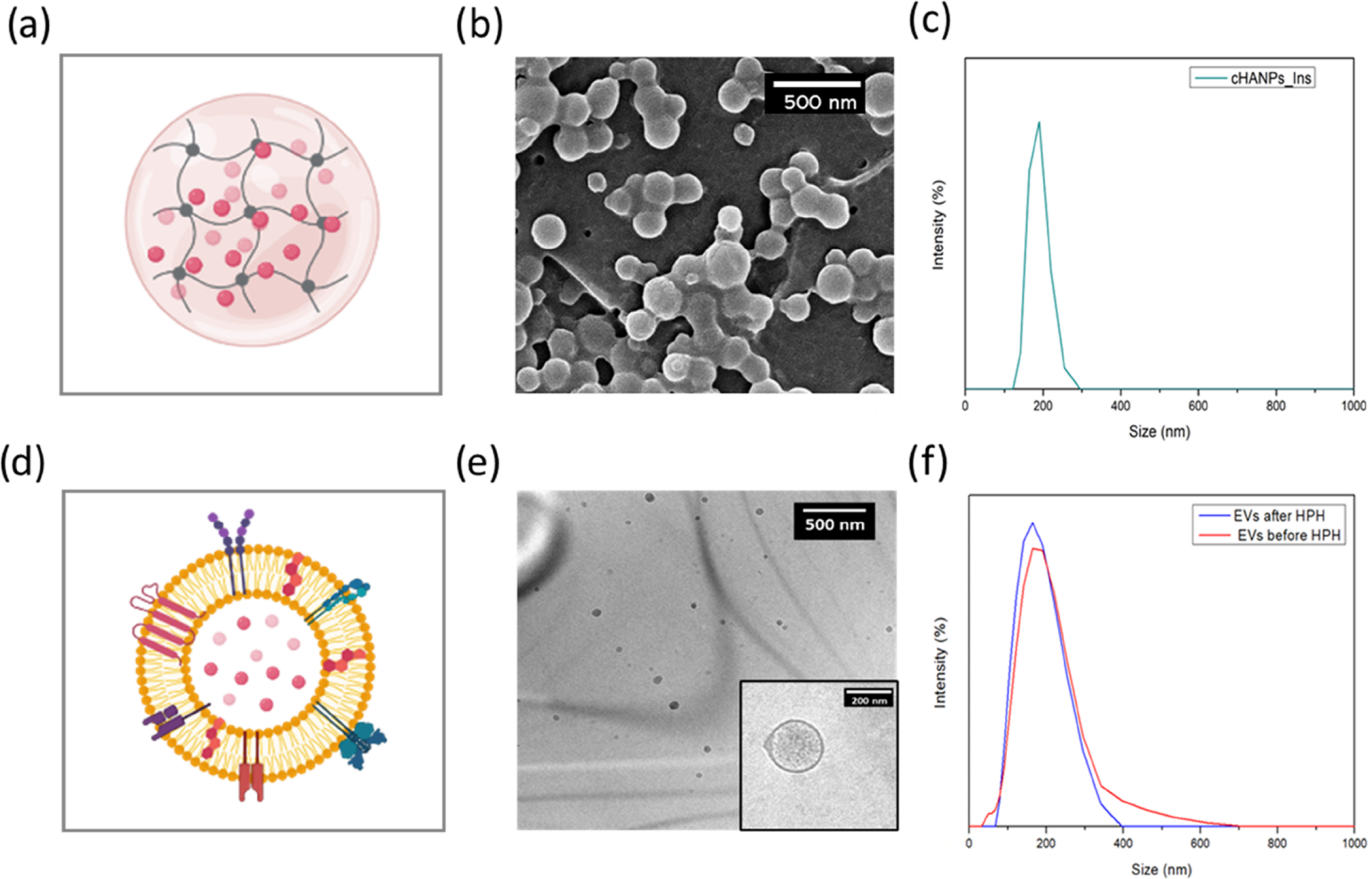
Synthetic identity of cHANPs and EVs. (a) Schematic illustration of Ins-cHANPs; (b) scanning electron microscopy (SEM) image of Ins-cHANPs; (c) particle size distribution (PSD) of Ins-cHANPs by dynamic light scattering; (d) schematic illustration of Ins-EVs; (e) cryo-TEM of Ins-EVs; and (f) PSD of EVs before and after insulin encapsulation by dynamic light scattering.

Cross-linked hyaluronic acid nanoparticles (cHANPs) are produced by microfluidics through nanoprecipitation in a consolidated hydrodynamic flow focusing (HFF) approach described in the dedicated Methods section and previously reported.^7,8,19^ Specifically, for insulin encapsulation, the solvent phase is composed of HA (0.05% w/V) and insulin (0.1 mg/mL), a pH of 12.3 is obtained by NaOH addition to promoting the cross-linking reaction with DVS, and it is injected at 27 μL/min in the middle channel of an X-junction chip. Acetone and the cross-linking agent DVS (4% V/V) are injected through the side channels of the X-junction at 110 μL/min. In Figure 1a, a schematic illustration of the architecture is presented. Figure 1b,c reports the morphologies obtained by scanning electron microscopy (SEM) observations and particle size distribution (PSD), as measured through dynamic light scattering. Results present a uniform population of spherical particles with a mean size of 186.3 ± 25.5 nm. The ζ potential measurement shows that cHANPs encapsulating insulin (Ins-cHANPs) are characterized by a slightly negative charge of −27 mV. To quantify cHANPs, EE% of the drug is chemically released from nanoparticles swollen by overnight exposure to NaOH alkaline solution and filtered, as described in the dedicated Methods section. A BCA assay is then performed on the lysed sample at different dilutions revealing an encapsulated amount of protein of 56 μg, corresponding to an encapsulation efficiency of 28.2% (Table S1). Circular dichroism (CD) measurements are also performed on processed insulin to confirm its structural stability upon encapsulation, Figure S1.

Extracellular Vesicles (EVs) are isolated from the culture medium of U87 cells, as described in the dedicated Experimental Section and as previously reported.^20^ Insulin encapsulation is performed through a high-throughput process recently proposed by our group, based on dynamic high pressure that significantly increases EVs’ permeability and results in temporary deformability of their membrane, thus improving the loading of active agents in these vesicles. A schematic illustration of insulin-encapsulating EVs (Ins-EVs) is presented in Figure 1d.

Due to high-pressure conditions, a study about the effect of insulin exposure to high pressure, with particular regard to the preservation of the hormone secondary structure, is performed by CD. The pressure and number of cycles have been ranging from 500 to 1500 bar and from 2 to 10, respectively. Results are presented in Figure S2 and demonstrate that the maximum number of cycles that assure insulin structure preservation decreases with increasing pressure, as expected. Matching the previous findings about the effect of pressure on the structural stability of EVs^20^ with the acquired data about insulin stability in HPH, the optimal process condition is set to 500 bar for 5 cycles. Figure 1e shows the morphology of the encapsulated vesicles by cryo-TEM, revealing their stability at high-pressure processing. This finding is confirmed by DLS analysis in Figure 1f presenting the PSD of EVs before and after treatment in the HPH, showing slight or no differences in the two samples, with Ins-EV displaying a mean size of 171.6 ± 55.81 nm. To measure the effective drug encapsulation in EVs, the sample collected from HPH is purified from excess insulin by a centrifuge at 1000 rpm, for 25 min at 10 °C in a spin Corning with 100 kDa cutoff. The purified Ins-EVs are then disrupted using 0.075% v/v Triton X 100 overnight followed by filtration. However, the presence of Triton X 100 interferes with both the UV spectrum of insulin and BCA assay measurement (data not shown). Thus, as presented in the Methods section, an indirect quantification is performed, measuring the amount of insulin purified from the encapsulated sample by BCA, revealing that 1.35 μg/mL is encapsulated with an EE% above 90%.

Table 1 summarizes data about the synthetic identity of both Ins-cHANPs and Ins-EVs.

**Table 1:** Physicochemical Properties of Ins-cHANPs and Ins-EVs

	size [nm]	ζ potential [mV]	loaded insulin [μg/mL]	EE%
Ins-EVs	171.6 ± 55.81	–14	35	92%
Ins-cHANPs	186.3 ± 25.5	–27	56.44	28.2%

### 3.2 Nanoparticle Uptake by Hep-G2 Adherent Cells by Flow Cytometry

**Figure 2:**
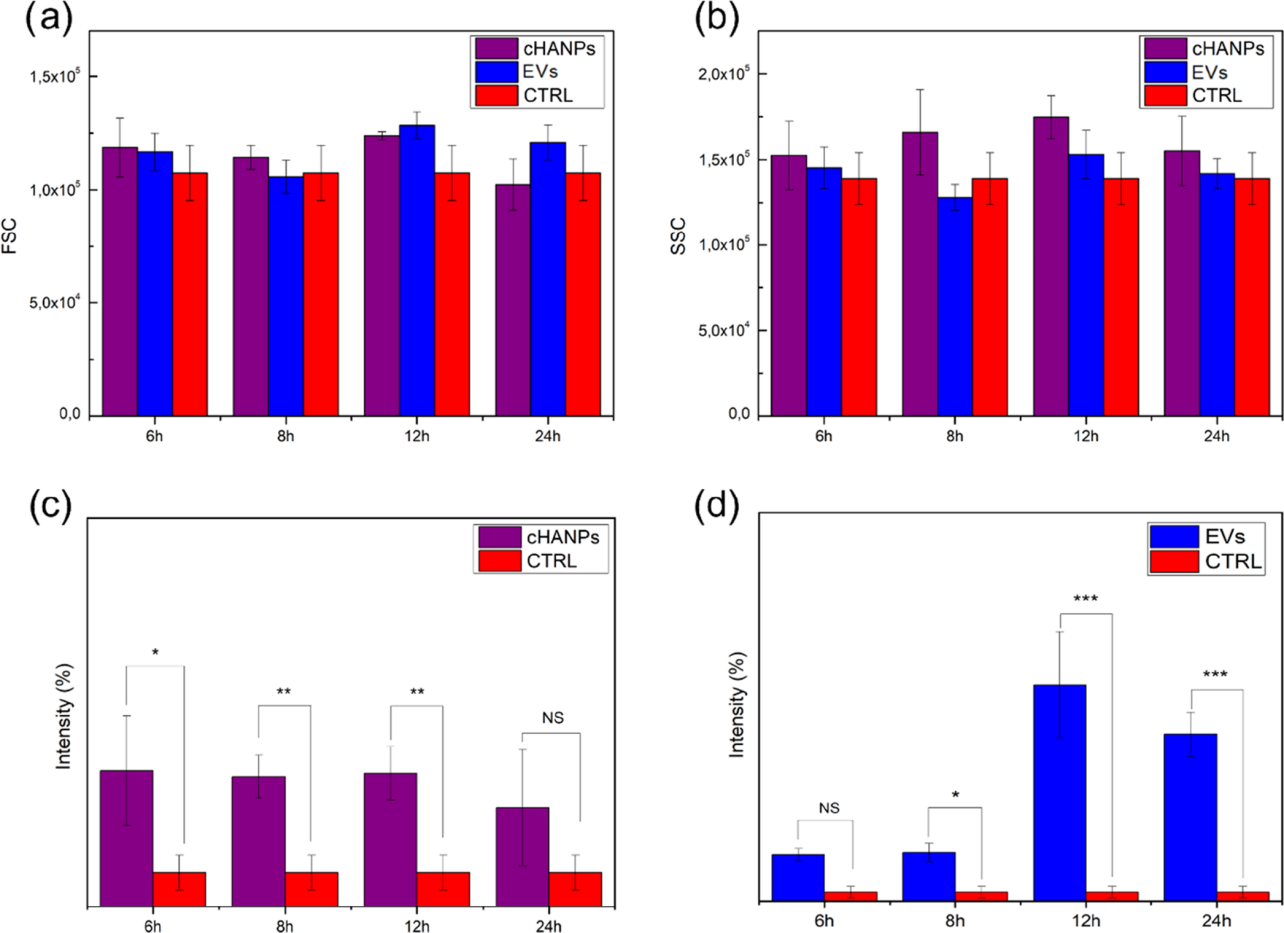
In vitro uptake of cHANPs and EVs by Hep-G2 cells. (a) Forward scattering (FSC) of EVs and cHANPs; (b) side scattering (SSC) of EVs and cHANPs; (c) fluorescent intensity of cHANPs internalizing Hep-G2 cells; and (d) fluorescent intensity of EVs internalizing Hep-G2 cells; (*) denotes *P* values <0.5, (**) *P* values <0.1, (***) *P* values <0.05, and NS indicates no significant differences.

The first level of interaction of the developed drug delivery systems with the biological environment is evaluated by studying the uptake of nanoparticles by adherent Hep-G2 liver carcinoma cells. Uptake is studied by flow cytometry measurements, which allows quantifying nanoparticle internalization by single-cell analysis. For this purpose, both fluorescently labeled cHANPs and EVs are produced. cHANPs encapsulating the standard NIR dye ATTO 633 are produced in microfluidics, as reported in the dedicated Methods section. Differently, the EV membrane is stained with the green lipid dye PKH67 for incubation. Fluorescently labeled nanoparticles (0.6 × 10^10^ particles/mL for cHANPs and 2.15 × 10^10^ particles/mL for EVs) are incubated with adherent cells and left to interact for 6, 8, 12, and 24 h. The analysis of the forward scattering (FSC) in Figure 2a reveals that no significant reduction in the measured signal with respect to the control can be detected for any sample at any time point as an indication of unaltered cell viability.^23,28^ Side scattering (SSC) as a measure of cell granularity is reported to indicate cell rearrangement as a consequence of nanoparticle uptake.^28^ As shown in Figure 2b, this value is significantly higher than the control for cHANPs at any time point, linearly increasing over time until the maximum is reached at 12 h. Similarly, the fluorescence intensity of cHANPs is higher than the control after 6 h of incubation, supporting the result that a significant uptake of nanoparticles occurred, as reported in Figure 2c. Differently, EVs produce a more modest increase in SSC intensity, despite a significant increase of fluorescence present from 12 h on, as reported in Figure 2d. At 24 h, a slight reduction both in SSC and the fluorescence intensity for both systems is detected as a result of the cell duplication causing the redistribution of fluorescence in daughter cells.

A direct comparison of the fluorescence intensity of cells exposed to cHANPs and EVs reveals that the increase in fluorescence due to EVs with respect to the control is several-fold higher than the increase produced by cHANPs. However, this cannot lead to conclude that a much higher number of EVs is taken up with respect to cHANPs since there are fundamental differences in the staining of the two samples. First, cHANPs and EVs deliver different concentrations of different dyes. Second, ATTO 633 is loaded into the cHANPs so emitting fluorescence from the inner core of the hydrogel, while PKH67 as a lipid dye stains the surface of EVs, producing direct emission of fluorescence in the suspending medium. We can observe instead that the uptake of cHANPs is already saturated after 6 h of incubation, where the maximum fluorescence intensity is detected. This is not surprising since Hep-G2 cells are reported as CD44-expressing cells, and HA is a natural substrate of these receptors. Therefore, HA-CD44 interaction might have favored the particle uptake. Differently, EVs reach the maximum of internalization up to 12 h with an exponential increase between 8 and 12 h. However, these results do not correlate with an increase in the SSC signal, supporting the hypothesis of EV fusion with the cell membrane. Indeed, despite a relevant increase in fluorescence, cell granularity is not significantly altered by a fusion mechanism as a possible result of unmodified cell internal reorganization of the cytoskeleton and organelles.^17^ Moreover, EV internalization does not appear to be saturated as the quantity of internalized particles increases, thus showing no dependence on the concentration gradient of particles across the membrane. These differences highlight the peculiarity of the uptake of each nanoparticle system, based on the different synthetic identities that trigger completely different uptake mechanisms, the characteristic time of interaction, and cell response.

### 3.3 Nanoparticle Penetration in 3D Liver-μTs

The nano-bio-interaction triggered by cHANPs and EVs is also investigated by analyzing their transport properties in 3D Liver-μTs as a reliable three-dimensional model for the study of nanoparticle tissue diffusion. Indeed, in the developed 3D configuration, the cells maintain the typical histotypic features and phenotypic characteristics of a specific tissue with relevant cell–cell interactions and epithelial polarization recapitulating the key morphological and functional properties of that tissue.^22,29^

Liver-μTs are produced as described in the dedicated Methods section and as previously reported. Briefly, they are produced using dynamic cells seeding on GPMs in a spinner flask bioreactor. The stirring to which the cells are subjected in the spinner flask determines cell proliferation and epithelial polarization on the GMP scaffold giving a three-dimensional appearance similar to the native liver.^27^ We investigate the morphological features of the 3D model via histological analysis. 3D Liver-μTs exhibited a homogeneous cell distribution around the microbead surface of the μTs, as reported in the hematoxylin- and eosin-stained section (Figure S3). Interestingly, cells maintained the typical liver-like histotypic features with cuboidal hepatocytes with a distinguishable polarized nucleus of the cells in purple (hematoxylin stains), and their cytoplasm in pink (eosin stain) with tight cell–cell contacts at day 7 of culture.

The study of nanoparticle uptake by adherent cells presented in the previous section allows us to define an optimal time window of interaction for each system with Hep-G2 cells. Considering the most complex environment of the 3D tissue, the time that might be required to observe the process of NP diffusion in the whole thickness of the tissue and to find a common observational time frame for the two systems, 24 and 48 h set as sampling time points for determination of NP interaction with and penetration in 3D μTs. As for the 2D uptake, the penetration study is performed in μTs cultured without serum to avoid interferences with EV internalization and to assure that the results obtained could be directly attributed to the NP synthetic identity and not to the possible formation of a protein corona.

**Figure 3:**
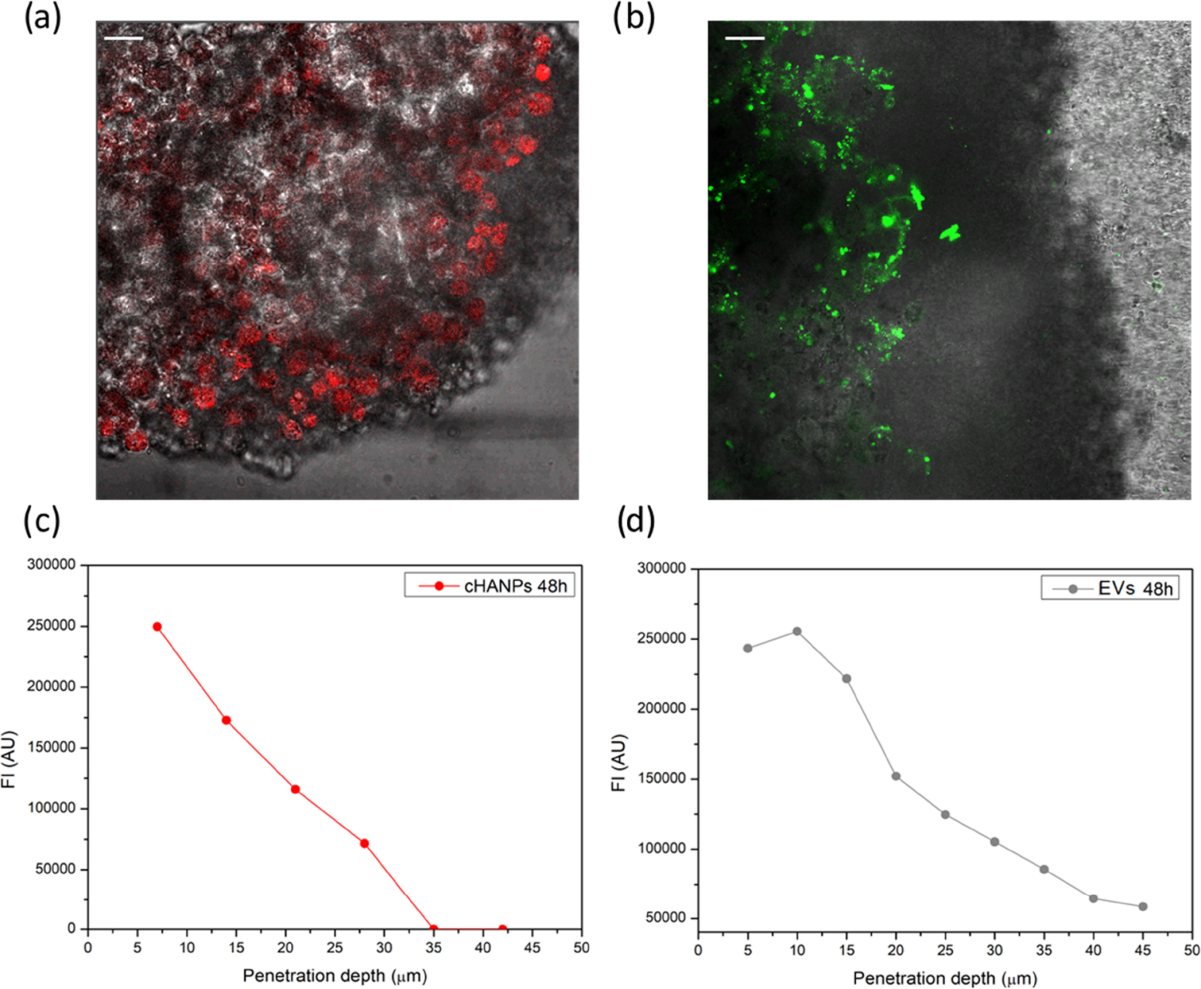
Penetration of cHANPs and EVs in liver-μTs. (a) Confocal Image of cHANPs in a liver μT; (b) confocal image of EVs in a liver μT; (c) quantification of cHANP penetration over liver μT thickness; (and d) quantification of EV penetration over liver-μT thickness. Scale bar 25 μm.

Thus, cHANPs (1.2 × 10^10^ particles/mL) and EVs (6 × 10^10^ particles/mL) are incubated with μTs and, after 24 h and 48 h, observed under the confocal microscope, as described in the dedicated Methods section. Figures 3a,b shows a significant presence of NPs in the core of the observed microtissues. From the images, it is possible to observe the absence of diffuse fluorescence as a sign of stability of dye encapsulation in both particle systems, an indirect indication of particle structural preservation at any time point, as shown in Figure S4 reporting confocal images acquired at both 24 and 48 h. For cHANPs, Figure 3a reveals that fluorescence can be detected in the whole cell cytosol, making the cells composing the tissues clearly visible. After 24 h, a significant NP uptake is detected, which might be attributed to the expression of CD44 receptors on Hep-G2 cells,^30^ as previously discussed, Figure S4. About EVs, Figure 3b shows a significantly different interaction of nanoparticles with the tissues. EVs accumulate in specific spots, interacting specifically with some domains. Whether these domains are located in specific regions of the cell membrane is not easy to be assessed. Other authors reported this behavior by identifying EV interaction with the cell membrane through quenching techniques in different cell lines.^15,31,32^

To further characterize the impact of nanoparticle synthetic identity on the depth of particle penetration inside the tissues, z-stacks are acquired, and the total image fluorescence is quantified for every z-step, and then plotted against tissue thickness (further details in the dedicated Methods section). Results in Figure S5a reveal that 24 h represents a sufficient time to allow cHANP penetration up to 45 μm deep in the tissue and, moreover, particles can diffuse in the tissues and preserve their stability until reaching inner-core cells. Figure 3c presents data obtained for 48 h, showing higher values on the surface of the tissue and significantly reduced fluorescence in the core of the tissue, reaching no increase in fluorescence with respect to the control above a thickness of 35 μm. Both profiles reveal that 24 h is not enough to saturate the uptake of the incubated cHANPs that continue increasing over time, as revealed by high superficial fluorescence at 48 h.

On the contrary, this fluorescence drop along the increasing thickness could reveal an occurring degradation process of NPs in the microtissue that causes a reduction of nanoparticle ‘diffusion.

Regarding EVs, the intensity of the total image fluorescence plotted against tissue thickness in Figure S5b shows that 24 h is not enough to guarantee a significant accumulation of the particles in the tissue. Indeed, at 48 h, fluorescence is significantly increased, although it seems that EVs are still majorly accumulated in the tissue surface with the higher fluorescence observed between 5 and 20 μm of tissue depth, Figure 3d.

Although direct comparison between fluorescence measurements in different tissues is not possible because of high variability in the local tissue morphology, results about nanoparticle penetration are supported by the analysis of other l-μTs presented in Figure S6, confirming the penetration profiles here presented.

Overall, these results highlight how a different synthetic identity determines an entirely different behavior of nanoparticles in biological environments and that this difference becomes more evident as the number of observed phenomena increases (i.e., tissue diffusion and cell uptake).

### 3.4 Therapeutic Implication of Nanoparticle Synthetic Identity: Effect of Insulin-Loaded Nanoparticles on Glucose Uptake by Liver-μTs

**Figure 4:**
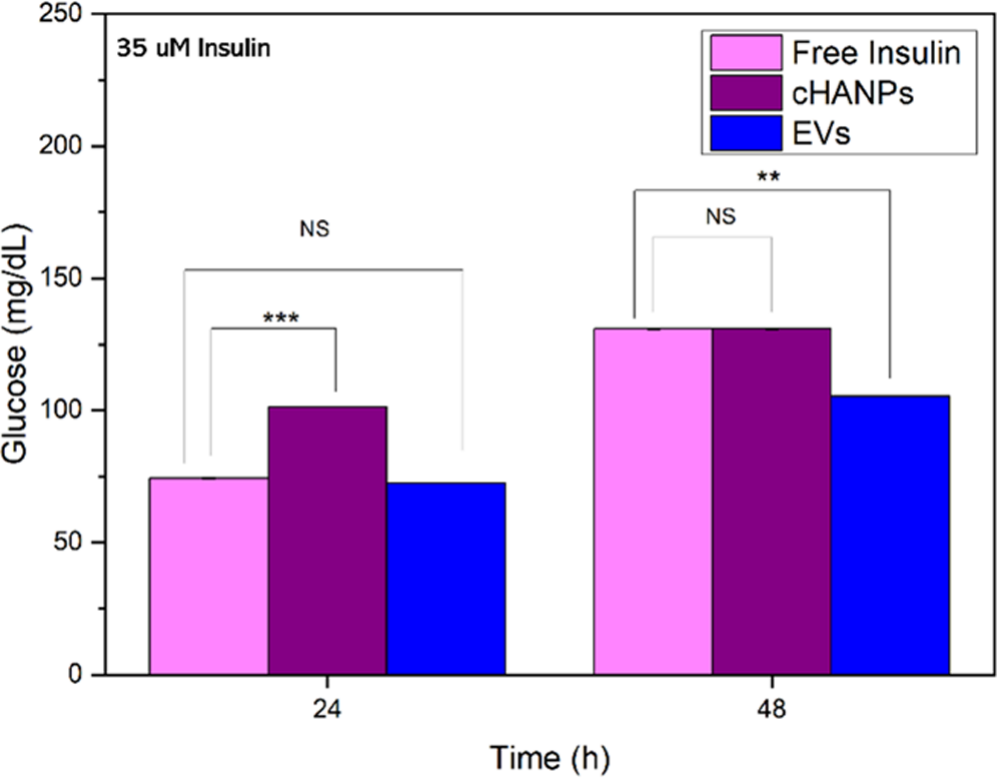
Quantification of glucose concentration in suspending culture medium of l-μTs treated with differently formulated insulin at 35 μM; (**) *P* values <0.1 and (***) *P* values <0.05, NS indicates no significant differences.

The previous sections clarified how the use of different delivery systems triggers different biological interactions in cellular uptake and tissue diffusion mechanisms. The comprehension and investigation of these interactions are of paramount importance since they inevitably reflect on the drug delivery system’s effectiveness and directly impact the outcome of the diagnostic or therapeutic function exerted by the vector *in vivo*. In this regard, the aim of this section is to highlight the implication of different nano-bio-interactions on the therapeutic effect of insulin as the model drug. To this purpose, we quantify the glucose taken up by liver-μTs over time and investigate how this consumption is altered when tissues are exposed to the same concentration of differently formulated insulin. In detail, 35 μM insulin as a free drug or encapsulated in cHANPs (Ins-cHANPs) and in EVs (Ins-EVs) is exposed to μTs, and glucose still available in suspending culture medium is quantified after 24 and 48 h of exposure. Results are presented in Figure 4 and reveal that Ins-EVs cause the highest reduction in glucose concentration in suspending culture medium already after 24 h of exposure and that this reduction becomes significantly higher after 48 h. The same effect could not be detected for cHANPs, producing slight or no improvements with respect to free insulin being only able to equal the result obtained for the free insulin, probably taken up by diffusion, in 48 h. These results highlight how the two systems mediate the drug’s interaction with its receptor at the cell membrane level. As previously speculated, EVs probably fuse with the cell membrane. This mechanism might offer a direct availability of the drug to its receptor, producing a faster and longer-lasting reduction of glucose concentration. On the other hand, cHANPs taken up by endocytosis somehow delay the drug/receptor interaction with respect to free insulin.

## 4 Conclusions

Two different delivery systems, hydrogel-based NPs (cHANPs) and lipid-based natural EVs, have been investigated to highlight how their different uptake mechanisms can trigger insulin activation in 3D L-μTs. Our results clearly state how crucial the optimal design of a drug delivery system for an intended biological task is, as the effectiveness of a specific active agent can be inevitably compromised when the inappropriate design is chosen. Indeed, we showed that Ins-EVs mediate the hormone interaction with the insulin receptor, favoring a faster and more pronounced glucose consumption with respect to free insulin, while Ins-cHANPs can only equal the glucose reduction caused by free insulin. We assume that this effect is due to the nano-bio-interaction triggered by the different bio-architectures; as previously reported by other authors, we also hypothesize that Ins-EVs fuse with the cell membrane of Hep-G2 cells, while Ins-cHANPs are polymer nanoparticles, generally taken up by endocytosis. In conclusion, this work highlights the specific set of nano-bio-interactions triggered by a full polymer, nanohydrogel-based vector and a natural, full-lipid EV, both delivering insulin in liver adherent cells and 3D highly reliable tissue models. This knowledge will help to evaluate the impact that different synthetic identities have on the final effectiveness of a formulation contributing to the field.

## Supporting Information Available

BCA results for insulin quantification in Ins-cHANPs (Table S1); CD of insulin at varying pressures and cycles (Figure S1); histological characterization of 3D liver-μTs (Figure S2); nanoparticle uptake by liver-μTs (Figure S3); penetration profile of cHANPs and EVs in l-μTs (Figure S4); and extra penetration profile of cHANPs and EVs (Figure S5) (PDF)
Microtissue uptake_cHANP_trasmission (Video V1); microtissue uptake EVs (Video V2); microtissue uptake of cHANP fluorescence (Video V3); microtissue uptake of cHANPs_merged (Video V4) (ZIP)
