# Supplementary material for: Insulin Activation Mediated by Uptake Mechanisms: A Comparison of the Behavior between Polymer Nanoparticles and Extracellular Vesicles in 3D Liver Tissues
Source: Biomacromolecules. 2023 Apr 6;24(5):2203–12. doi: 10.1021/acs.biomac.3c00102 (PMC10170511; doi:10.1021/acs.biomac.3c00102)
Supplement: Supplementary file 1 — bm3c00102_si_001.pdf [file bm3c00102_si_001.pdf]

Title

Insulin Activation Mediated by uptake mechanisms: a comparison between polymer nanoparticles and extracellular vesicles behavior in 3D liver tissue

Authors

Angela Costagliola di Polidoro[1,2 ¥], Zahra Baghbantarghdari[2 ¥], Vincenza De Gregorio[1,4], Simona Silvestri[1,2,3], Paolo Antonio Netti[1,2,3], Enza Torino[1,2,3]*

Affiliations

[1] Interdisciplinary Research Centre on Biomaterials (CRIB), University of Naples Federico II, P.le Tecchio 80, Naples, 80125, Italy

[2] University of Naples Federico II, Department of Chemical, Materials and Production Engineering (DICMaPI), P.le Tecchio 80, 80125, Naples, Italy

[3] Fondazione Istituto Italiano di Tecnologia, IIT, Largo Barsanti e Matteucci 53, 80125, Naples, Italy

[4] Department of Biology, University of Naples "Federico II", Complesso Universitario di Monte S Angelo, Naples, Italy

First, pure insulin solution at neutral and alkaline pH is studied by BCA assay showing no significant differences in the absorbance values measured (data not shown). So, it is concluded that while measuring the concentration of insulin, there is no negative interference of pH in the measurement, at least in the operative range of pH from 7.4 to 12.5. In the following Table S1 BCA assay results from measurement of lysed cHANPs at two different dilutions are presented.

Table S1. BCA results from measurement of lysed cHANPs for Insulin quantification

	Mean Abs	Measured Concentration [µg/mL]
Ins-cHANPs 1:3	342.7	8.265
Ins-cHANPs 1:6	185.7	3.95

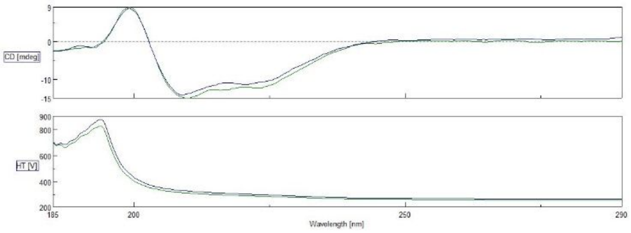

Figure S1. Evaluation of insulin stability at operative conditions for cHANPs production. Overlapping of CD spectra of Insulin at pH 7.5 (in green) and pH of 12.1 (in blue)

Insulin stability and particularly, preservation of its secondary structure at high pressures in the HPH is studied by circular dichroism (CD). Insulin is treated at different pressure values ranging from 500 to 1500 mbar for different number of cycles ranging from 1 to 10. Results in Figure S1 show the CD measurements for the maximum number of cycles that, fixed the pressure, assure insulin secondary structure preservation. Secondary structure is measured for reference insulin and for treated insulin. As expected, the maximum number of preservative cycles decreases with increasing pressure.

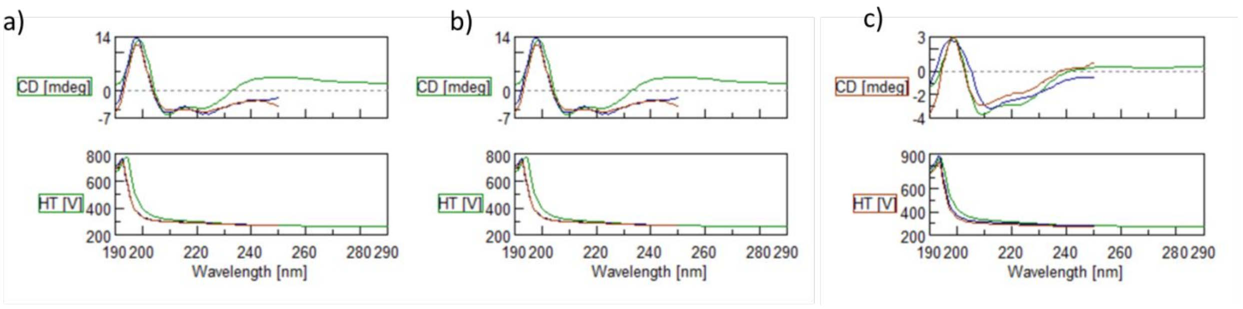

Figure S2. Impact of pressure and number of cycles on secondary structure of Insulin by CD. a) Insulin treated at 500 mbar for 9 cycles; b) Insulin treated at 1000 mbar for 4 cycles; c) Insulin treated with 1500 mbar for 2 cycles. Reference insulin, post process insulin and insulin in residual volume post purification are reported in green, blue and red respectively.

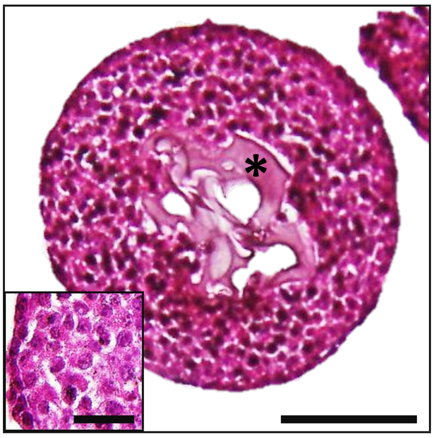

Figure S3. Histological characterization of 3D Liver µTs. Representative image and high-resolution inset display hematoxylin and eosin staining of 3D Liver µTs at 7 days of culture (nucleus in purple and cytoplasm in pink); the asterisk (*) indicates Gelatin Porous Microbeads (GMP). Scale bar, 100 µm and 50 µm (inset).

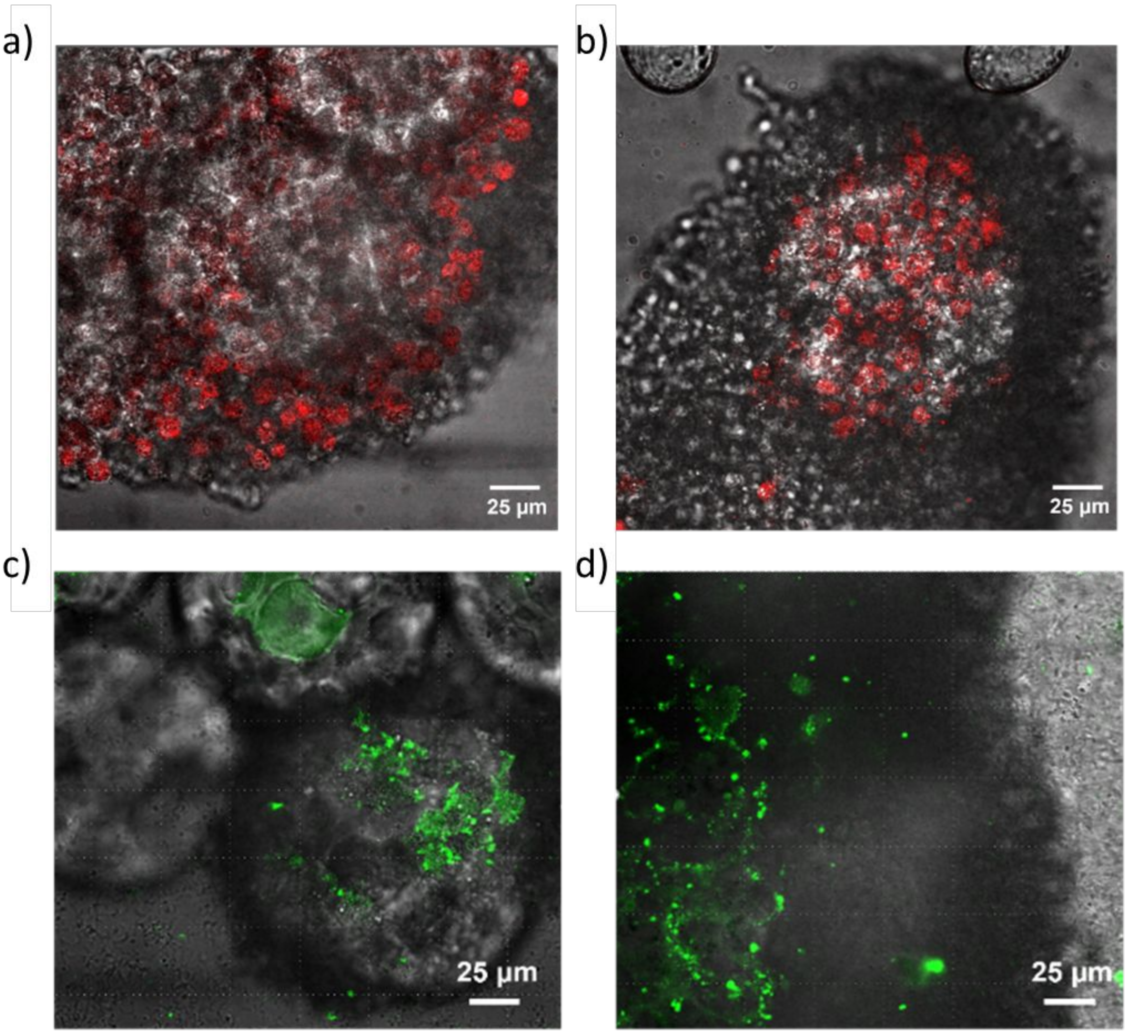

Figure S4. Nanoparticle uptake by Liver µ-Ts. a) cHANPs uptake after 24h incubation; b) cHANPs uptake after 48h incubation; c) EVs uptake after 24h incubation; d) EVs uptake after 48h incubation.

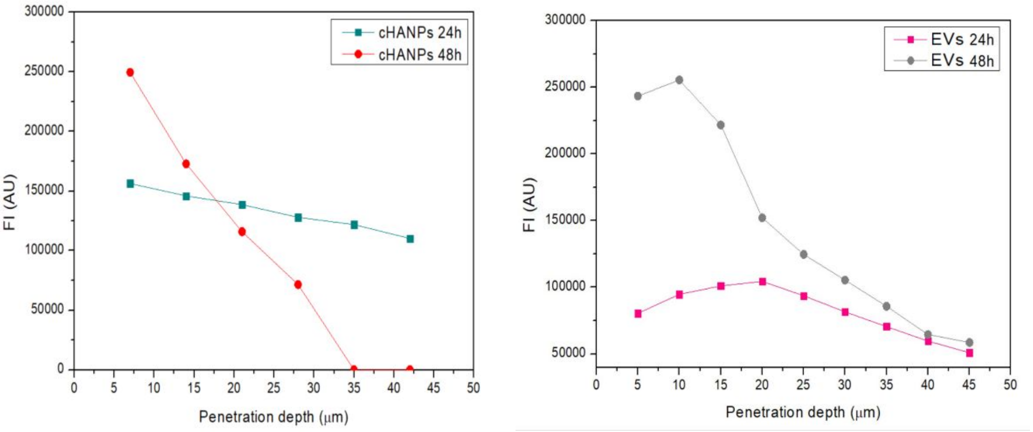

Figure S5. Penetration profile of cHANPs and EVs in L-µTs in Figure S3

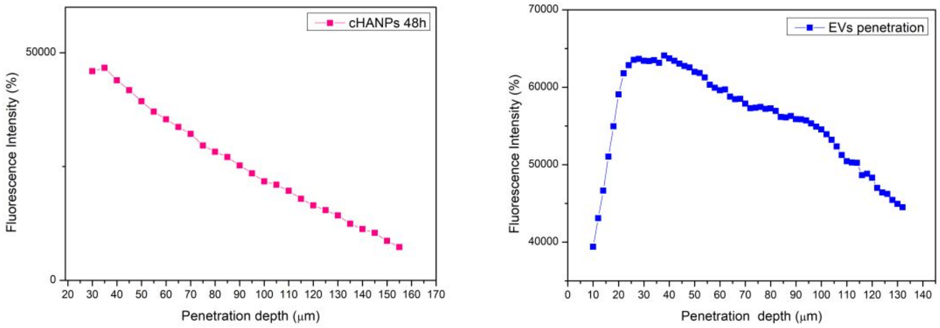

Figure S6. Penetration profile of cHANPs and Exosomes at 48h in µTs in supplementary videos. a) penetration profile of cHANPs as presented in video V1 b) penetration profile of EVs as presented in video V2.
